# The complete mitochondrial genome of *Gadus chalcogramma* and phylogenetic analysis

**DOI:** 10.1080/23802359.2018.1462118

**Published:** 2018-04-12

**Authors:** Han-Kyeol Sim, Jeong-Nam Yu, Deuk-Hee Jin

**Affiliations:** a Department of Marine Molecular Bioscience, Gangneung-Wonju National University, Gangneung, Korea;; b Biodiversity conservation and Change Division, Freshwater Biodiversity Research Bureau, Nakdonggang National Institute of Biological Resource (NNIBR), Sangju-Si, Korea

**Keywords:** *Gadus chalcogramma*, mitochondrial genome, phylogenetic

## Abstract

Here, we report the complete mitochondrial genome sequence of Alaska Pollock, *Gadus chalcogramma*. The genome sequence was obtained via long PCR reactions using universal primer sets for the fish mitochondrial genome. The total length of mitochondrial genome was 16,571 bp, consisting of 13 protein-coding genes, 22 tRNA genes, 2 rRNA genes, and one control region (D-loop). Except for ND6 and eight tRNA genes, all of the other mitochondrial genes were encoded on the heavy strand. The NJ tree of the combined 13 protein-coding gene sequences of *Gadus chalcogramma* possesses a relatively closer relationship with the Okhotsk Sea Pollock (*Gadus chalcogrammus*). Our complete mitochondrial genome will be valuable resources for phylogeny, genetics, and conservation of the *Gadus chalcogramma* in Korea.

## Main text

**Figure 1.**
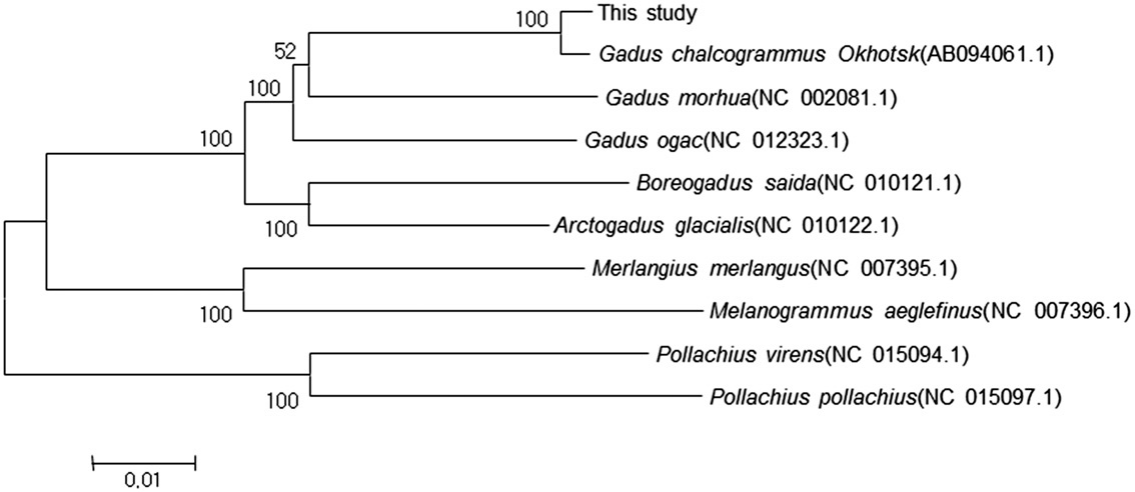
Phylogenetic tree of *Gadus chalcogramma* and related species’ complete mtDNA. Neighbour-joining tree based on the 13 protein-coding genes of genus *Theragra chalcogramma*. The numbers at the nodes are bootstrap values computed using 10,000 replications and Kimura’s 2-parameter distance model. The scale bar indicates 0.01 substitutions per nucleotide position.

Walleye or Alaska Pollock, *Gadus chalcogramma* is a commercially important species on both sides of the North Pacific and the Bering Sea. Particularly, in Korea, this species is an economically important fish as a traditional food. Recently, populations of this species have been declining dramatically in Korea due to overfishing and environmental changes during the last three decades: the highest catches occurred in 1981, followed by continuous decrease through the 1990s, ending with a complete collapse of the population in the 2000s (Kang et al. 2013). However, molecular genetic studies have not been conducted of Walleye or Alaska Pollock in Korea. Therefore, we report the complete mitochondrial genome of *G. chalcogramma* as a first step to elucidate genetic characteristics of this species in Korea. The voucher specimen (Frozen tissue and DNA) was collected from the Goseong-ayajin port (N 38°16'22.01", E 128°33'22.58") at Gangwon Province of Korea. It was stored in the fish collections in the Laboratory of Marine Molecular Biology  at Gangneung-Wonju National University, Korea. The obtained mitochondrial genome sequences were long and accurate; PCR reactions using universal primer sets were designed for fish mitochondrial genome (Miya and Nishida 1999). We have delegated the analysis to Biomedic Corp. and the method used for primer walking. The sequences were assembled, aligned, and annotated using MEGA6.0 (Tamura et al. 2013) and tRNAscan-SE 1.21 (Lowe and Eddy 1997). The sequence of *G. chalcogramma* with the annotation of genes were deposited in GenBank under the accession number of MH018252. The complete mitochondrial genome was similar to that of most other vertebrates (Chen et al. 2012; Li et al. 2012). It was 16,571 bp in length, containing 13 typical vertebrate protein coding genes, 22 transfer RNA genes, 2 ribosomal RNA genes, and a control region. Except for ND6 and eight tRNA genes, all of the other mitochondrial genes are encoded on the heavy strand. The AT content in whole genome and PCGs is 57.6% and 58.0%, respectively. The majority of protein-coding genes (PCGs) started with ATG, with one exception of COI starting with GTG. Four types of stop codons including three complete codons, TAA, TAG, AGG and AGA for vertebrate were mostly used (Ojala et al. 1981; Boore 1999). Seven overlapping areas were observed, including four notable overlapping positions between protein coding genes (ATP8 and ATP6, ATP6 and COX3, ND4L and ND4, and ND5 and ND6), which have also been reported for other fish and vertebrate species (Boore 1999). The 22 tRNA genes ranged from 66 to 75 bp, and most of the tRNAs could be folded into typical cloverleaf secondary structures. The control region (CR, 849bp) located between tRNA-Pro and tRNA-Phe had not repeated particular nucleotides but showed the presence of poly-T and poly-A. The neighbour-joining gene tree of 11,466 bp of the combined 13 protein-coding gene sequences of family Gadidae with significant bootstrapping value. Our data was most closely related to *G. chalcogrammus* Okhotsk (AB094061.1) (Figure 1). These data provide useful information for further understanding of the phylogenetic classification and evolution of Gadidae species and the genetic structure of *G. chalcogramma*. Moreover, these findings could be used as the basis of the genetic conservation management of *G. chalcogramma* in Korea.
